# Spectral-domain optical coherence tomography combined with electroretinography in the assessment of conbercept for neovascular age-related macular degeneration: a preliminary study

**DOI:** 10.3389/fnins.2023.1179421

**Published:** 2023-04-25

**Authors:** Xing Wang, Peng Wang

**Affiliations:** Chongqing Key Laboratory of Ophthalmology, Department of Ophthalmology, Chongqing Eye Institute, The First Affiliated Hospital of Chongqing Medical University, Chongqing, China

**Keywords:** neovascular age-related macular degeneration, multifocal electroretinogram, full-field electroretinogram, spectral-domain optical coherence tomography, conbercept

## Abstract

**Objective:**

To observe the effect of three consecutive intravitreal injections of conbercept in the treatment of neovascular age-related macular degeneration (nAMD), to investigate the correlation between retinal anatomy and retinal function by spectral-domain optical coherence tomography (SD-OCT) and electroretinography (ERG), to evaluate the short-term clinical efficacy of conbercept in the treatment of nAMD, and to explore the value of ERG as a predictor of treatment efficacy.

**Method:**

A retrospective investigation was conducted on 36 patients (36 eyes) treated with intravitreal injections of conbercept at 0.5 mg a month for three consecutive courses. Data collected included the best corrected visual acuity (BCVA), central retinal thickness (CRT), retinal pigment epithelium (RPE) elevation volume in 1 mm-diameter (1RV), 3 mm-diameter (3RV), and 6 mm-diameter circles around the fovea (6RV), amplitude density and latency of the P1 wave in the multifocal electroretinography (mf-ERG) R1 ring and amplitude and latency in full-field electroretinography (ff-ERG) at baseline and monthly. The paired t test was used to compare the difference between pre- and posttreatment. Pearson correlation analysis was used to analyze the correlation between macular retinal structure and function. The difference was significant when *p* < 0.05.

**Results:**

At 12 weeks, the BCVA, CRT, 1RV, 3RV, 6RV, the P1 wave amplitude density of the mf-ERG R1 ring and the ff-ERG amplitude parameters were all significantly improved (*p* < 0.001). The BCVA in logMAR was positively correlated with CRT; 1RV, 3RV, and 6RV were negatively correlated with the amplitude density and latency of the mf-ERG R1 ring P1 wave. There were no severe ocular or systemic complications during the follow-up period.

**Conclusion:**

Conbercept is useful for the short-term treatment of nAMD. It can safely improve the visual acuity of affected eyes and restore the structure and function of the retina. ERG could serve as an objective indicator of function for evaluating the efficacy of and determining the need for retreatment during nAMD treatment.

## Introduction

Neovascular age-related macular degeneration (nAMD) is a common blinding disease manifested as chronic and progressive aging of the macula (Ambati and Fowler, 2012). Several factors are associated with the development of nAMD, including age, environment, smoking, lifestyle, genetics (ultraviolet radiation-induced) chronic photodamage, hypertension, metabolic disorders, oxygen damage, and inflammatory reactions (Sakurada et al., 2013).

In recent years, a series of imaging examinations such as spectral-domain optical coherence tomography (SD-OCT), fundus fluorescein angiography (FFA), indocyanine green angiography (ICGA), multifocal electroretinography (mf-ERG) and full-field electroretinography (ff-ERG) have been widely used in ophthalmology (Castillo et al., 2014). SD-OCT is a high-resolution, 3D reconstruction-capable examination that can display the retinal tissue structure at different layers, measure the thickness of the retina and its different layers, accurately locate retinopathy, and be subjected to multilevel analysis (Wilde et al., 2015). Visual electrophysiological examinations are commonly used to evaluate various clinical macular diseases, these objective, specific and noninvasive examinations can also be used for the early detection of subclinical and undetected dysfunction (Renner et al., 2005). Invented by Sutter and Tran (1992) in the early 1990s, mf-ERG is a technique for objectively assessing visual cell function in the macula by obtaining the local retinal response to different stimulation units simultaneously. Its main advantages are its ability to identify lesions and assess disease severity (Moschos and Nitoda, 2018).

Anti-vascular endothelial growth factor (VEGF) is among the priority therapies for the treatment of nAMD. The efficacy and safety of anti-VEGF agents in eliminating choroidal neovascularization (CNV) have been clearly demonstrated. Anti-VEGF agents can effectively improve visual acuity, reduce blindness, and improve quality of life (Funk et al., 2009; Scott et al., 2015). Among anti-VEGF agents, conbercept is China’s first novel biological drug, mainly targeting nAMD (Li et al., 2014). Conbercept is a fully human-derived protein that binds tightly to VEGF-A, VEGF-B, and placental growth factor (PIGF) (Lu et al., 2015). With high affinity and long half-life, it effectively inhibits the proliferation and migration of vascular endothelial cells and angiogenesis (Cui et al., 2018). Additionally, the unique structure of conbercept prolongs its effective duration of action and exposure, reducing the number and risk of injections and easing the burden of treatment (Nguyen and Guymer, 2015).

The criteria for evaluating the efficacy of anti-VEGF therapy and the need for retreatment rely on OCT, FFA, ICGA, mf-ERG, and other methods, such as microperimetry (Rai et al., 2021) and pupillographic objective perimetry (Rai et al., 2022). The only functional indicator currently in use is vision loss of more than five letters, which is subjective. Additionally, many hospitals do not have Early Treatment Diabetic Retinopathy Study (ETDRS) charts. Therefore, we intend to combine changes in best corrected visual acuity (BCVA), SD-OCT, and ERG to evaluate the clinical efficacy of conbercept in the treatment of nAMD and to understand the correlation between retinal anatomy and retinal function. We also sought to assess the value of ERG in anti-VEGF therapy and determine whether it could serve as an objective indicator of function in evaluating the efficacy of treatment and determining the need for retreatment.

## Methods

### Research design

This study is a retrospective clinical observation conducted according to the tenets of the Declaration of Helsinki. Thirty-six subjects (36 eyes) diagnosed with nAMD by fundus examination, SD-OCT, FFA, and ICGA were recruited from June 2017 to October 2018 from the Department of Ophthalmology of the First Affiliated Hospital of Chongqing Medical University, Chongqing, China. All patients received an intravitreal injection of 0.5 mg conbercept once a month for three consecutive courses. The injections were performed in strict accordance with the clinical trial data of conbercept (Zhang et al., 2011; Li et al., 2014). The inclusion criteria were as follows: (1) age ≥ 50 years, BCVA 0.05–0.5, and the nAMD diagnostic criteria (Adrean et al., 2018); (2) FFA/ICGA suggesting the presence of macular neovascularization; and (3) no previous treatment (e.g., triamcinolone, anti-VEGF, photodynamic therapy). Anyone with one of the following conditions was excluded: (1) allergy to local anesthetics, mydriatic eye drops, and contrast agents; (2) moderate refractive opacity that affects imaging, aphakic eyes; (3) history of internal eye surgery, trauma or previous fundus laser photocoagulation; (4) glaucoma or high intraocular pressure; (5) fundus diseases such as pathological myopia, diabetic retinopathy, retinal vein occlusion, proliferative vitreoretinopathy, MH, idiopathic or autoimmune uveitis; and (6) scleromalacia.

### Assessment

BCVA was assessed with the international logarithmic visual acuity scale. Intraocular pressure (IOP) was measured by a noncontact automatic tonometer (NIDEK NT-510, Japan). Slit-lamp examination and fundus photography (TOPCON TRC-NW8 fundus camera, Japan), SD-OCT, and ERG were performed at baseline and 4 weeks after each intravitreal injection. FFA and ICGA were performed at baseline by a Heidelberg Spectralis HRA illuminator. At baseline and at each visit, we carefully examined the anterior segment (including the cornea, anterior chamber, iris, lens), vitreous chamber, and IOP and recorded the opacity of the refractive media. We also tested the subject for infection or uveal inflammatory reactions before and after intravitreal injection and, if present, assessed and recorded the severity of the reactions.

SD-OCT was performed with a spectral-domain OCT instrument (SPECTRALIS HRA OCT, Heidelberg, Germany). The overlapping mode was used for the same patient for each measurement. Central retinal thickness (CRT) was delineated as the average thickness of the neurosensory retina within a central 1 mm-diameter area. The retinal pigment epithelium (RPE) layer strips were manually fine-tuned to obtain a more accurate measurement. The lesion volume within 1 mm-, 3 mm-, and 6 mm-diameter circles around the macular fovea was measured using macular topographic analysis software. The CRT and the RPE elevation volume in the 1 mm-diameter (1RV), 3 mm-diameter (3RV) and 6 mm-diameter circles around the macular fovea (6RV) were recorded.

ERG was performed with a RETI-Port/Scan 21 system (Roland Consult Gmbh, Wiesbaden, Germany). Prior to the examination, 0.5% topiramate ophthalmic solution was administered to fully dilate the pupil to approximately 7 mm in diameter, and cocaine hydrochloride ophthalmic solution was given for surface anesthesia. Reference electrodes were attached to each side of the crotch, and recording electrodes were hung on the conjunctiva surface of the lower third of the eyes and then fixed with tape. The patient’s pupil was tested for dilatation according to the standards of the International Society for Clinical Electrophysiology of Vision (Berezovsky et al., 2003). Dark-adapted 0.01 ERG (rod response), dark-adapted 3.0 ERG (combined response), dark-adapted 3.0 oscillatory potentials, light-adapted 3.0 ERG (cone response), and light-adapted 3.0 flicker response (cone-pathway response) were recorded. mf-ERG was performed in 61 stimulation unit modes. Since the amplitude density of the P1 waves mainly corresponds to the distribution range of visual cells, with the highest density at the fovea and gradually decreasing toward the periphery, the measured R1 ring mainly represents the retinal function of the fovea (Chen et al., 2005). The mf-ERG recordings were thus analyzed by measuring the amplitude density and latency of the P1 wave of the R1 ring. During the recording, abnormalities such as electrode detachment, baseline drift, and interference were observed to avoid shifting of eye position. All examinations were completed by the same physician.

### Statistical analysis

We used SPSS 22.0 software (SPSS, IBM, Armonk, NY) for statistical analysis. The mean ± standard deviation represents the measurements. All data were verified to be normally distributed using the Kolmogorov–Smirnov test. The means of the BCVA, CRT, 1RV, 3RV, 6RV, and the mf-ERG (the amplitude density and latency of the P1 wave of the R1 ring) and ff-ERG parameters (amplitude and latency of the dark-adapted 0.01 b wave, dark adapted 3.0 a/b wave, dark-adapted 3.0 oscillatory potentials, light-adapted 3.0 a/b wave and light-adapted 3.0 flicker responses b wave) were compared between baseline and 12 weeks. We used the two paired-samples *t* test for all comparisons. Correlations between the BCVA and SD-OCT, mf-ERG, and ff-ERG parameters were analyzed using the Pearson correlation. *p* < 0.05 indicated statistical significance.

## Results

### Patient characteristics

The average age of the 36 subjects was 71.28 ± 9.16 years. Among them, 19 (52.78%) were male, and 17 (47.22%) were female. There were 15 (41.67%) cases of nAMD in the right eye and 21 (58.33%) cases in the left eye. Other details are available in Table 1. In our study, all 36 patients had monocular nAMD.

**Table 1:** Baseline characteristics of patients with AMD.

*N*		36
Age, *y*		71.28 ± 9.16
Sex	Male, *n*	19 (52.78%)
	Female, *n*	17 (47.22%)
Side	Right, *n*	15 (41.67%)
	Left, *n*	21 (58.33%)
Duration of AMD, *m*		6.28 ± 2.76
MNV Type I/II/III, *n*		15/19/2

AMD, age-related macular degeneration; MNV, macular neovascularization; n, number; m, month. Values are mean ± SD.

### BCVA

Twelve weeks after the first injection, the mean BCVA (0.85 ± 0.34) improved by 2–3 lines (0.56 ± 0.29), and the difference from baseline was statistically significant (*t* = 8.6524, *p* = 3.2 × 10^–10^) (Table 2).

**Table 2:** Changes in BCVA, fundus findings, and SD-OCT after intravitreal injection of Conbercept.

	Baseline (0 week)	4 weeks	8 weeks	12 weeks	End vs. Pre	
					*T* value	*p* value
BCVA (logMAR)	0.85 ± 0.34	0.69 ± 0.30	0.58 ± 0.30	0.56 ± 0.29	8.6524	3.2 × 10^−^10
CRT (μm)	472.14 ± 181.53	389.5 ± 126.57	332.19 ± 102.43	311.61 ± 95.67	7.4002	1.2 × 10^−8^
1RV (mm^3^)	0.36 ± 0.14	0.31 ± 0.11	0.26 ± 0.07	0.25 ± 0.08	6.6614	1.0 × 10^−7^
3RV (mm^3^)	3.04 ± 0.98	2.59 ± 0.71	2.23 ± 0.65	2.17 ± 0.72	7.0254	3.5 × 10^−8^
6RV (mm^3^)	8.17 ± 2.91	6.66 ± 2.42	5.36 ± 2.53	4.90 ± 2.49	7.9148	2.6 × 10^−9^
*Retinal*						
Hemorrhages (%)	66 ± 1.78	56 ± 1.23	42 ± 1.29	21 ± 1.09	7.4002	3.5 × 10^−8^
*Hard*						
Exsudates (%)	58 ± 1.12	54 ± 1.97	32 ± 1.22	27 ± 1.90	6.8924	2.1 × 10^−8^
IRC (%)	15 ± 2.21	14 ± 1.45	13 ± 1.34	10 ± 1.32	7.0981	2.5 × 10^−8^
SRF (%)	17 ± 1.01	15 ± 1.21	13 ± 1.11	9 ± 1.21	7.2223	1.9 × 10^−8^
PED (%)	22 ± 1.67	20 ± 0.97	17 ± 1.01	15 ± 0.87	6.5671	1.6 × 10^−8^
IHF (%)	29 ± 2.03	25 ± 1.78	23 ± 1.56	20 ± 1.21	7.1261	2.4 × 10^−8^
SHRM (%)	16 ± 1.77	14 ± 1.01	12 ± 1.71	9 ± 0.87	7.2212	2.6 × 10^−8^

BCVA, best corrected visual acuity; CRT, central retinal thickness; 1RV, 1 mm round in circle of RPE uplift volume; 3RV, 3 mm round in circle of RPE uplift volume; 6RV, 6 mm round in circle of RPE uplift volume; IRC, intraretinal cysts; SRF, subretinal fluid; PED, pigment epithelial detachment; IHF, intraretinal hyperreflective foci; SHRM, subretinal hyperreflective material. Values are mean ± SD.

### SD-OCT

**Figure 1:**
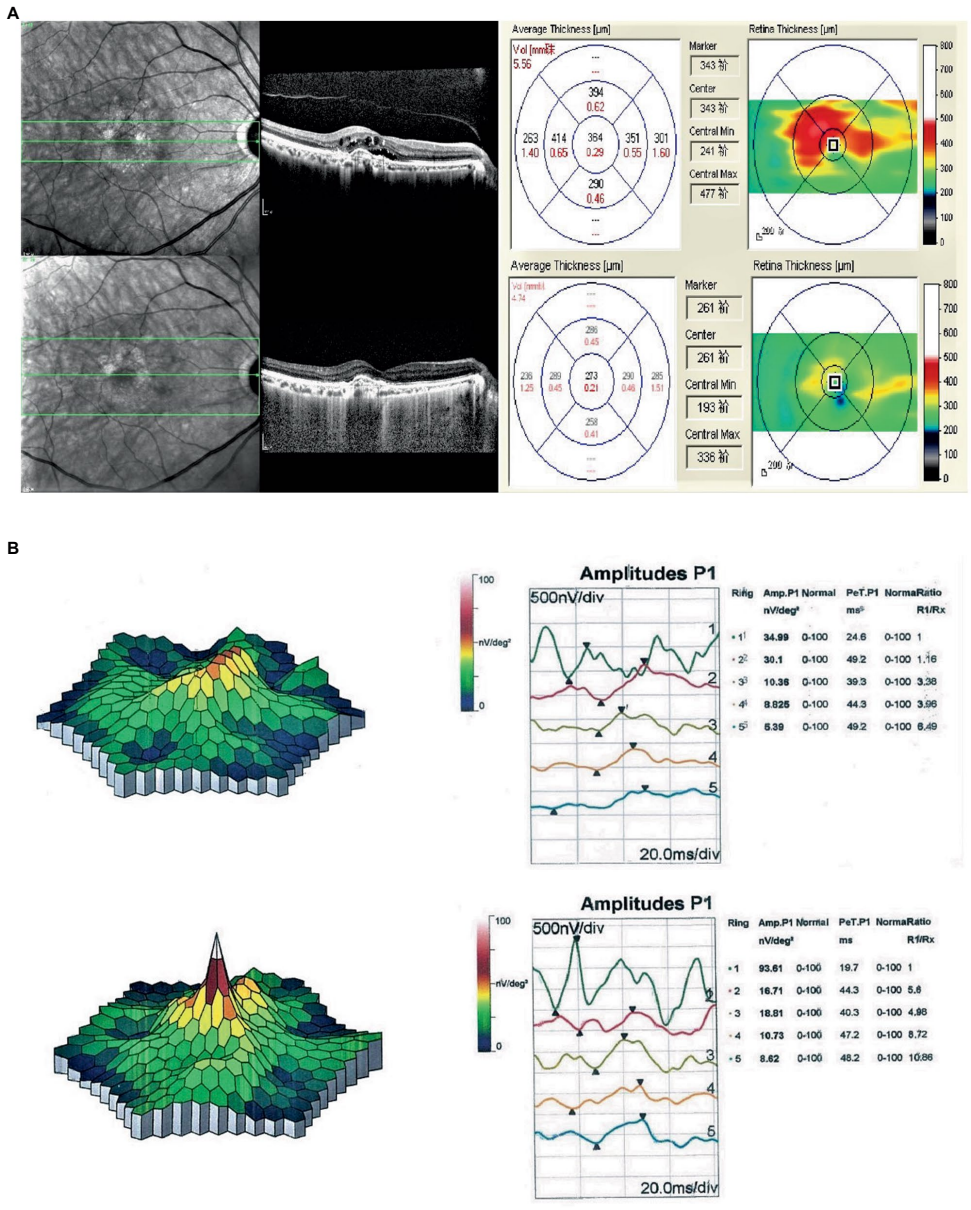
**(A)** Compared with the first OCT, OCT at the final follow-up for the nAMD patients essentially showed recovery of the macular area. The CRT decreased by 91 μm, the 1RV decreased by 0.08 mm^3^, the 3RV decreased by 0.58 mm^3^, and the 6RV decreased by 0.82 mm^3^. **(B)** Comparison of the first mf-ERG in nAMD patients at the last follow-up showed a significant increase in the P1 wave amplitude density of the R1 ring (58.62 nV/deg^2^), and no substantial change in the P1 wave latency.

The SD-OCT examinations showed that the 36 eyes had a loss of normal morphology of the macular area at baseline with intraretinal cysts, subretinal fluid (SRF), retinal thickening, pigment epithelial detachment (PED), subretinal hyperreflective material (SHRM) and intraretinal hyperreflective foci (IHFs). The specific percentages of these alterations are detailed in Table 2. The mean CRT (472.14 ± 181.53) significantly decreased to 311.61 ± 95.67 at 12 weeks (*p* = 1.2 × 10^–8^); the mean 1RV (0.36 ± 0.14) significantly decreased to 0.25 ± 0.08 at 12 weeks (*p* = 1.0 × 10^–7^); the mean 3RV (3.04 ± 0.98) significantly decreased to 2.17 ± 0.72 at 12 weeks (*p* = 3.5 × 10^–8^); and the mean 6RV (8.17 ± 2.91) significantly decreased to 4.90 ± 2.49 at 12 weeks (*p* = 2.6 × 10^–9^) (Table 2; Figure 1A).

### ERG

Comparison of the P1 wave data of the mf-ERG R1 ring at baseline (36.47 ± 14.25) and after 12 weeks (58.36 ± 13.45) indicated a considerable increase in amplitude density (*p* = 5.0 × 10^–11^) but not in the P1 latency (38.13 ± 9.75 vs. 39.21 ± 9.63, *p* = 0.22) (Table 3; Figure 1B). Comparisons of the ff-ERG parameters showed that all but latency significantly increased after 12 weeks. The details are shown in Table 3.

**Table 3:** Changes in the mf-ERG R1 ring and ff-ERG after intravitreal injection of Conbercept.

	Baseline (0 week)	12 weeks	End vs. Pre	
			*T* value	*p* value
mf-ERG R1 ring				
P1 amplitude density (nv/deg^2^)	36.47 ± 14.25	58.36 ± 13.45	−9.3533	5.0 × 10^−11^
P1 latency (ms)	38.13 ± 9.75	39.21 ± 9.63	−1.2568	0.22
ff-ERG				
Dark-adapted				
Scotopic b-wave amplitude (μV)	35.48 ± 27.31	79.49 ± 45.32	−7.6377	5.9 × 10^−9^
Scotopic b-wave latency (ms)	45.88 ± 4.80	46.33 ± 4.38	−0.8031	0.43
Combined maximal a-wave amplitude (μV)	89.46 ± 46.39	145.01 ± 65.02	−6.1812	4.46 × 10^−7^
Combined maximal a-wave latency (ms)	21.61 ± 2.26	22.56 ± 1.77	−3.1490	3.33 × 10^−3^
Combined maximal b-wave amplitude (μV)	165.49 ± 78.36	267.62 ± 99.07	−7.4794	9.29 × 10^−9^
Combined maximal b-wave latency (ms)	44.32 ± 3.51	46.23 ± 2.25	−3.2089	2.85 × 10^−3^
Oscillatory potentials amplitude (μV)	17.87 ± 12.53	24.50 ± 13.88	−3.7067	7.22 × 10^−4^
Oscillatory potentials latency (ms)	25.36 ± 0.69	25.55 ± 0.49	−1.8335	0.07
Light-adapted				
Cone response a-wave amplitude (μV)	41.45 ± 27.39	65.48 ± 39.06	−6.7436	8.21 × 10^−8^
Cone response a-wave latency (ms)	25.83 ± 4.51	26.72 ± 3.63	−1.6999	0.10
Cone response b-wave amplitude (μV)	22.42 ± 15.01	38.11 ± 20.91	−5.3081	6.31 × 10^−6^
Cone response b-wave latency (ms)	32.84 ± 3.54	33.38 ± 3.95	−1.4808	0.15
30-Hz flicker amplitude (μV)	57.71 ± 23.95	79.93 ± 39.83	−3.9114	4.03 × 10^−4^
30-Hz flicker latency (ms)	67.14 ± 15.43	67.81 ± 16.17	−0.2912	0.77

mf-ERG, multifocal electroretinogram; ff-ERG, full-field electroretinogram. Values are mean ± SD.

### Correlation analysis

Pearson correlation analysis was conducted between the BCVA (logMAR) and OCT and ERG measurements at baseline and 12 weeks separately for all 36 nAMD eyes, and the corresponding scatter plots were generated. The analyzes indicated that the BCVA was positively and significantly correlated with the CRT, 1RV, 3RV, and 6RV, negatively and significantly correlated with the amplitude density and latency of the P1 wave of the mfERG R1 ring and negatively but not significantly correlated with the ff-ERG parameters.

**Figure 2:**
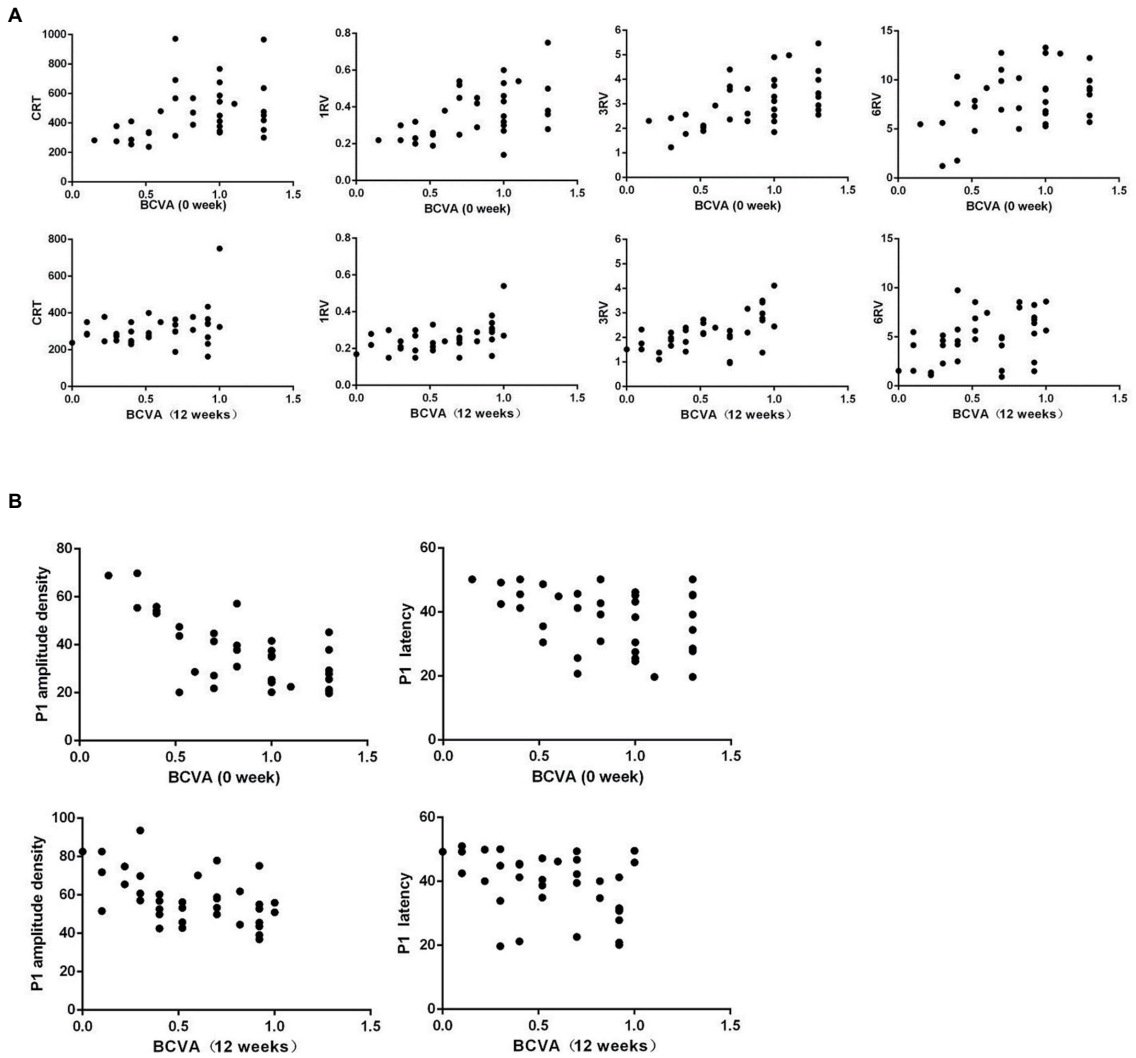
**(A)** Scatter plots of the first and last BCVA (logMAR) and OCT measurements for nAMD patients show a significant, positive correlation between the first BCVA (logMAR) and CRT, but the final, positive correlation is not significant. The first and last BCVAs (logMAR) is significantly, positively correlated with 1RV, 3RV, and 6RV. **(B)** The first and last BCVA (logMAR) and mf-ERG correlation scatter plots of nAMD patients show a significant, negative correlation between the BCVA (logMAR) and P1 wave amplitude density and latency.

Notably, at 12 weeks, the BCVA and CRT were positively correlated, but not significantly (*p* = 0.06) (Tables 4, 5; Figures 2A,B).

**Table 4:** Correlation analysis between BCVA (logMAR) and OCT measurements.

	Baseline (0 week)		12 weeks	
	*r* value	*p* value	*r* value	*p* value
CRT	0.3824	0.02	0.314501	0.06
1RV	0.4322	0.85 × 10^−2^	0.476404	0.33 × 10^−2^
3RV	0.5361	0.08 × 10^−2^	0.488476	0.25 × 10^−2^
6RV	0.3831	0.02	0.389903	0.02

CRT, central retinal thickness; 1RV, 1 mm round in circle of RPE uplift volume; 3RV, 3 mm round in circle of RPE uplift volume; 6RV, 6 mm round in circle of RPE uplift volume.

**Table 5:** Correlation analysis between BCVA (logMAR) and ERG measurements.

	Baseline (0 week)		12 weeks	
	*r* value	*p* value	*r* value	*p* value
mf-ERG R1 ring				
P1 amplitude density	−0.6774	5.68 × 10^−2^	−0.4953	0.21 × 10^−2^
P1 latency	−0.3432	0.04	−0.3729	0.03
ff-ERG				
Dark-adapted				
Scotopic b-wave amplitude	−0.2348	0.17	−0.0524	0.76
Scotopic b-wave latency	−0.3090	0.07	−0.4729	0.36 × 10^−2^
Combined maximal a-wave amplitude	−0.3018	0.07	−0.3938	0.02
Combined maximal a-wave latency	−0.1657	0.33	−0.4621	0.45 × 10^−2^
Combined maximal b-wave amplitude	−0.1050	0.54	−0.2886	0.09
Combined maximal b-wave latency	0.0107	0.95	0.1376	0.43
Oscillatory potentials amplitude	0.2297	0.18	0.2136	0.21
Oscillatory potentials latency	−0.0724	0.67	−0.1641	0.35
Light-adapted				
Cone response a-wave amplitude	0.0791	0.65	0.0764	0.66
Cone response a-wave latency	−0.0011	0.99	−0.2671	0.43
Cone response b-wave amplitude	−0.2903	0.09	−0.5707	0.03 × 10^−2^
Cone response b-wave latency	−0.1953	0.25	0.1678	0.34
30-Hz flicker amplitude	0.0466	0.79	−0.1043	0.55
30-Hz flicker latency	−0.1128	0.51	−0.0024	0.99

mf-ERG, multifocal electroretinogram; ff-ERG, full-field electroretinogram.

### Complications and adverse reactions

The IOP was within the normal range (10 to 21 mmHg) in all 36 eyes before and after treatment during the follow-up period; any variations in the IOP were small and nonsignificant (*p* > 0.05). Two eyes with corneal epithelial defects and four eyes with subconjunctival hemorrhage after injection eventually recovered. None of the 36 eyes had severe ocular or systemic complications (e.g., secondary glaucoma, iatrogenic cataract, vitreous hemorrhage, endophthalmitis, retinal tears). None of the patients had even mild inflammation.

## Discussion

Currently, we still do not fully understand nAMD. Untreated patients are prone to central visual distortion, fixed black shadow, and central visual impairment (Sikorav et al., 2017). Older age at diagnosis, poor vision, high severity, and longer duration of the disease are strongly associated with poor prognosis (Nguyen et al., 2018). A variety of factors lead to the development of nAMD, which is mainly characterized as the formation of CNV via the action of multiple cytokines (such as VEGF, angiopoietin, inflammatory factors, insulin-like growth factor1, cyclooxygenase-2, chemokine receptor-3, and primary fibroblast growth factor). Any of these cytokines causes damage to Bruch’s membrane, which in turn is accompanied by a relative or absolute increase in VEGF and aggregation of inflammatory factors, leading to the occurrence of CNV (Cheung et al., 2017; Chen et al., 2018).

Studies have shown that the formation of CNV is mainly due to the high expression of VEGF (Klettner et al., 2013; Liu et al., 2017). VEGF-A, VEGF-B, VEGF-C, VEGF-D, and PIGF are five members of the VEGF family. VEGF is essentially a glycoprotein, which is a type of polypeptide growth factor (Tarallo and De Falco, 2015). Under hypoxic and inflammatory conditions, VEGF increases plasminogen activator (PA) activity by increasing the mRNA expression of PA and plasminogen activator inhibitor-l (PAI-1), resulting in denaturation of the extracellular matrix, increased vascular permeability, vascular endothelial cell migration, blood–retinal barrier impairment, proliferation and neovascularization (Shibuya, 2013). Fundus manifestations usually include retinal drusen, frequent bleeding, exudation, and oedema. The bleeding further results in scar development, leading to the irreversible loss of central vision (Zhang and Ma, 2007). Anti-VEGF products such as bevacizumab, ranibizumab, aflibercept, and conbercept all act to reduce VEGF activity, bringing hope to nAMD patients (Yazdi et al., 2015; Moreno et al., 2016; Avery et al., 2017; Liu et al., 2019). Among them, conbercept is a new generation of fusion protein drugs developed independently in China that can fully bind VEGF-A isoforms, VEGF-B and PIGF, with higher affinity than aflibercept and bevacizumab (Yu et al., 2012; Ventrice et al., 2013). It effectively reaches all layers of the fundus and has a long half-life. Fifteen days after injection, the retinal concentration of conbercept is still 1,000 times higher than retinal VEGF exposure, allowing extension of the injection interval and reducing the financial burden for the patient (Zhang et al., 2009). Some multicenter, large sample randomized controlled clinical registry studies (HOPE, AURORA, PHOENIX) have confirmed the superiority and safety of the multitarget antagonistic effects of conbercept (Zhang et al., 2011; Li et al., 2014; Liu et al., 2019).

In this study, after 36 patients received three core treatments with conbercept, the BCVA demonstrated 2 to 3 rows of improvement over the baseline visual acuity (*p* < 0.001), suggesting that conbercept can effectively stabilize and improve vision in the treatment of nAMD. Furthermore, the CRT, 1RV, 3RV, and 6RV decreased significantly, confirming the specific effect of conbercept on reducing the retinal thickness and lesion volume in the macula. These findings indicate that conbercept can inhibit CNV leakage, promote fluid absorption, and restore normal retina morphology, which provides theoretical support for the improvement of BCVA from the anatomical structure. ERG, a noninvasive, highly specific and highly sensitive examination, can objectively reflect the function of the retina (Robson et al., 2018). Maturi et al. (2006) studied the amplitude density of macular function on mfERG in 9 patients with nAMD before and one month after bevacizumab treatment. The results showed a significant improvement in the electrophysiological response of the macula after treatment, suggesting the need to assess the degree of recovery of visual function in conjunction with mf-ERG indicator when treating patients with nAMD. The study claimed that in early AMD the rods degenerate earlier than the cones and that the decrease in rod-mediated photopic sensitivity is more pronounced than that in cone-mediated photopic sensitivity (Jackson et al., 2002; Feigl et al., 2005). We found that the amplitude density of the P1 wave of the mf-ERG R1 ring and the amplitude of the ff-ERG parameters increased significantly following conbercept treatment, while the ERG latency did not change significantly from baseline. These increases are thought to be the result of partial recovery of visual cell function. However, the latency on ERG reflects the speed of visual cell propagation, which varies from site to site. Due to the lack of regularity in the distribution of nAMD lesions, latency does not accurately reflect visual cell function impairment. Combining the results for the BCVA and the OCT and ERG parameters, we conclude that conbercept is helpful for the short-term treatment of nAMD.

The BCVA in logMAR was significantly positively correlated with the SD-OCT measurements but negatively correlated with the P1 wave measurements of the mf-ERG R1 ring. This is consistent with previous studies showing that the mf-ERG response objectively predicts future vision loss and has a high sensitivity in assessing the effects of intravitreal injections of anti-VEGF agents (Pedersen et al., 2010; Wu et al., 2014; de Oliveira Dias et al., 2017). In our study, one eye (without PED) showed no significant changes in the BCVA or P1 wave density after three courses of treatment but did show significant decreases in the CRT, 1RV, 3RV, and 6RV; this may be related to the long course of the disease. In nAMD, some of the visual cell functions are permanently lost and cannot be restored; consequently, there was no significant improvement in visual acuity even after the anatomy improved. This suggests that the morphological changes revealed by SD-OCT do not fully equate with the changes in visual cell function. Gonzalez-Garcia et al. (2016) found that mf-ERG can detect electrophysiological changes in the retina prior to vision loss during AMD. This suggests that mf-ERG is more sensitive in diagnosing and monitoring disease progression and changes than OCT, although both OCT and mf-ERG can be effective in this regard. Similarly, Ambrosio et al. (2015) noted that mf-ERG allows a more direct assessment of macular function than other measures. In AMD, it shows high specificity and sensitivity in detecting early retinal dysfunction and could serve as a predictor of vision loss. Other studies have reported that ff-ERG can be used as a functional tool to assess the retina after intravitreal injections (Skaat et al., 2011; Andrade et al., 2016). Our results showed an increased amplitude in ff-ERG parameters but no significant correlation with the BCVA. We believe this may have occurred due to our sample size and short follow-up period.

In addition, the clinical application of microperimetry has further expanded the psychophysical study of retinal function in recent years. Similar to ERG, microperimetry can detect retinal function through light stimulation in different regions and of varying intensity (Meleth et al., 2011). Similar to ERG, microperimetry can be used to assess visual dysfunction in the reticular pseudodrusen (RPD) in the macula. Forte et al. (2014) found that compared with those with early AMD, patients with only RPD changes in the fundus and normal vision had decreased visual sensitivity on microperimetry. However, in patients with mid-term atrophic AMD, changes in RPD did not affect the visual sensitivity on microperimetry. The RPD plays an important role in the progression of AMD. In the future, further studies of the effects of RPD on atrophic AMD and visual function using microperimetry are needed (Querques et al., 2014).

In conclusion, to our knowledge, our investigation is the first report on BCVA combined with ERG and SD-OCT to evaluate the therapeutic efficacy of conbercept in nAMD. In the short term, conbercept can safely and significantly improve and stabilize vision in the treatment of nAMD, which contributes to the functional and structural recovery of the macula. Our sample size was limited, and only three treatments were analyzed. More multicenter, large-sample, prospective, randomized clinical controlled trials are needed to explore the long-term efficacy, drug safety, treatment regimen, and course of treatment of conbercept.

Our investigation showed that BCVA, SD-OCT, and ERG can be combined to morphologically and functionally assess the condition of the eye. The ability to use the results obtained by ERG and further predict subsequent functional deterioration could be valuable in developing treatment protocols and designing clinical trials. Further research is needed to predict the prognostic value for nAMD and other macular diseases based on the ERG response. Although ERG is not currently used routinely in patient testing, a combined assessment would be more objective and comprehensive in assessing the clinical efficacy of conbercept for nAMD. During nAMD treatment, ERG could serve as an objective functional indicator for assessing treatment efficacy and determining the need for retreatment.

## Data availability statement

The original contributions presented in the study are included in the article/supplementary material, further inquiries can be directed to the corresponding author.

## Ethics statement

Ethical review and approval was not required for the study on human participants in accordance with the local legislation and institutional requirements. Written informed consent for participation was not required for this study in accordance with the national legislation and the institutional requirements.

## Author contributions

XW and PW contributed to the conception of the study. XW collected data and wrote the manuscript. PW extracted the data and revised the manuscript text. All authors reviewed the manuscript and approved it for publication.

## Conflict of interest

The authors declare that the research was conducted in the absence of any commercial or financial relationships that could be construed as a potential conflict of interest.

## Publisher’s note

All claims expressed in this article are solely those of the authors and do not necessarily represent those of their affiliated organizations, or those of the publisher, the editors and the reviewers. Any product that may be evaluated in this article, or claim that may be made by its manufacturer, is not guaranteed or endorsed by the publisher.
